# Hypertension risk pathways in urban built environment: the case of Yuhui District, Bengbu City, China

**DOI:** 10.3389/fpubh.2024.1443416

**Published:** 2024-09-18

**Authors:** Kangkang Gu, Yao Jing, Jingjing Tang, Xianjie Jia, Xinmu Zhang, Beichen Wang

**Affiliations:** ^1^School of Architecture and Planning, Anhui Jianzhu University, Hefei, Anhui, China; ^2^Anhui Institute of Land Spatial Planning and Ecology, Hefei, Anhui, China; ^3^Department of Epidemiology and Statistics, School of Public Health, Bengbu Medical College, Bengbu, Anhui, China

**Keywords:** built environment, hypertension, health behavior, impact path, mediating effects

## Abstract

**Introduction:**

The rapid development of urbanization has brought about changes in residents’ living environment and behavior, leading to health challenges such as hypertension. An improvement in the built-up environment in the community could contribute to the construction of a healthy city, promote the active life of the residents, and prevent and relieve hypertension. However, there is little research on the relationship between the built environment of the community and hypertension. This cross-sectional study aims to evaluate the relationship between communities’ built environment, health behavior, and hypertension grade of residents in Yuhui District of Bengbu City.

**Methods:**

This study is based on data from the 2022 Health Survey of Residents in 21 communities. To investigate the impact of the community’s built environment on residents’ hypertension and the underlying mechanisms, regression and structural equation modeling were employed.

**Results and discussion:**

The results show that the built environment of urban communities has a significant impact on the residents’ hypertension. The presence of high densities of supermarkets, convenience stores, parks and plazas, but low densities of clinics and hospitals, has been identified as a significant risk factor for the development of high blood pressure among the residents. Nevertheless, the adoption of healthy behaviors, including regular walking, physical activity, and a diet rich in fruit and vegetables, can play an important role in reducing the risk of hypertension. The findings of this study show that enhancements to the built environment in urban neighborhoods could contribute to a reduction in the prevalence of hypertension among residents. Furthermore, the implementation of efficacious health interventions in urban settings would facilitate the alteration of residents’ health behaviors and enhance their overall health status.

## 1 Introduction

Rapid urbanization is the major factor affecting urban public health. One notable consequence of urbanization is the marked increase in the incidence of hypertension. In some Asian countries, cardiovascular diseases have become the leading cause of mortality (1) and, in some developing countries, urban populations are more likely to suffer from diseases such as hypertension than rural populations (2). Pedestrian-friendly environments characterized by high densities, mixed-use functions, and accessible road networks have a significant inhibitory effect on reducing hypertension among the population in Europe and the United States. In China, community environments that are multi-functional, in addition featuring a dense road network, ample health facilities, and healthy food, are also helpful in reducing the incidence of hypertension (3). Related studies have shown that the urban built environment may influence the regulation of blood pressure among people (4). The prevalence of high population density in residential areas may impose constraints on the manner in which individuals engage in leisure activity (5). Concurrently, a reduction in public activity space and green open space *per capita* may result in increased psychological stress for residents, which increases the risk of hypertension. It is evident that atmospheric pollution (6) and noise pollution (7) contribute significantly to hypertension. This is particularly the case in developing countries, where the majority of roads are designed with the primary objective of facilitating the movement of motor vehicles, rather than providing safe and accessible routes for pedestrians. Consequently, the high-density road network has an adverse effect on the blood pressure of the population (8). Clinics and hospitals provide residents with more opportunities for blood pressure measurement and health education, giving them the possibility to prevent hypertension in advance (9). The proliferation of high-density supermarkets and convenience stores carry the potential of diminishing the availability of nutritious food items, such as fruits and vegetables. Conversely, this situation results in an increased intake of high-energy foods in the residents’ daily diets. Consequently, such a diet increases the likelihood of residents developing high blood pressure (10). Park plazas, being green open spaces and public activity spaces that promote people’s health, have been shown to be effective in reducing the risk of hypertension in a number of studies (11).

The hypertensive population (defined as residents with blood pressure levels higher than 120/80 mmHg) exceeds 20% in the Yuhui District in Bengbu City. In this area, the aging population is also approaching 30%. It is a compact community with an array of public facilities and is significantly distinct from other areas. Consequently, in order to examine the relationship between the built environment and health, and to elucidate the impact of the built environment on the health of the residents, Yuhui District in Bengbu City was selected as a representative case study. It has been demonstrated that the construction of compact neighborhoods enhances the accessibility of fitness venues. It is more likely to facilitate greater levels of physical activity among the residents (12) and mitigate the risk of hypertension. As such, effective spatial planning can reduce the risk of hypertension of the residents. Nevertheless, the number of studies examining the influence of the built environment on health pathways remains limited. Prior research has concentrated on the impact of residents’ travel and dietary habits on the risk of hypertension (13). The proximity of residents’ homes to their places of work has been demonstrated to reduce the frequency of car travel on a daily basis. This, in turn, has been shown to lead to an increase in the use of healthier modes of travel, such as walking and cycling. Consequently, these habits have a beneficial impact on the residents’ capacity to maintain optimal blood pressure levels. Residents living in neighborhoods with low availability of healthy foods are prone to consuming cheap but unhealthy food in nearby fast food restaurants, which can increase the risk of hypertension (14).

The majority of current research on the urban built environment and hypertension among residents is conducted at the macro-scale, with studies focusing on the county level (15), the provincial level (16), and the downtown areas of large cities (17). There is a paucity of research at smaller scales, such as the community level, and the impact of issues such as aging and older urban areas has been relatively neglected. Furthermore, the impact of individual health status and poor lifestyle choices is overlooked. Therefore, this study employs the Yuhui District in Bengbu City as a case study to examine the influence of the built environment on the residents’ hypertension and the communities’ associated behavioral patterns. Furthermore, this article presents recommendations regarding the configuration of urban spatial structure. It is hoped that the recommendations provided will enhance the efficacy of active health intervention in urban areas, reduce the risk of hypertension and other diseases, and improve the overall health of the residents.

## 2 Materials and methods

### 2.1 Overview of the study area

Bengbu, located in the northeast of Anhui Province, is an important city in the middle and lower reaches of Huaihe River; it’s also the central city in northern Anhui. The study area is 21 plots in 3 neighbourhoods in Yuhui District, Bengbu City, involving three communities: Chaoyang Street, Daqing Street, and Diaoyutai Street. According to the survey conducted in 2020, the permanent population of the study area was 120,000, and the aging population nearly account for 30%, making it a severely aging area. Concurrently, 20% of the population is afflicted with hypertension. The blood pressure levels of these individuals exceed the generally accepted norm (120/80 mmHg). This poses a significant risk of cardiovascular diseases among the population. Therefore, this area is selected as the research unit.

### 2.2 Data sources

The data on residents’ hypertension come from the survey of daily activities and health status of Bengbu residents carried out by the research group in 2022. Random household questionnaires were distributed among the 21 communities in Yuhui District and their response gathered. The survey staff received training and passed an examination. The questionnaire was reviewed by the ethics committee of Bengbu Medical College and was given to the residents only after obtaining their informed consent. The questionnaire includes personal basic information (gender, age, marital status, educational background, monthly income), daily behavior (daily walking time, physical exercise time, frequency of eating fruits and vegetables), health status (height, weight, medical expenses of the year) and bad habits (smoking and drinking). All questionnaires were subjected to a comprehensive review. A questionnaire was deemed invalid if more than 15% of the options selected by the respondent failed to provide a coherent representation of their views on the matter in question. A total of 2,539 questionnaires were distributed, and 2,412 were returned as valid, representing a 95% response rate. The blood pressure of the respondents was measured on the spot and the hypertension grade was calculated (18). In accordance with the recommendations of a medical practitioner from Bengbu Medical College, the subject group underwent blood pressure assessments between 8 and 10 a.m. on the same day. The blood pressure measurements were taken for a minimum of 5 min per individual. A minimum of two blood pressure readings were recorded for each measurement, with an interval of one to 2 min between readings. The average of the two readings was then calculated. The device utilized for the measurement of blood pressure was an Omron U702 sphygmomanometer. In accordance with the standards set forth by the World Health Organization, normal adult blood pressure is defined as 120 mmHg systolic and 80 mmHg diastolic. A diagnosis of hypertension was made if the subject exhibited a systolic blood pressure of ≥140 mmHg and/or a diastolic blood pressure of ≥90 mmHg on two separate occasions.

The data of the Built Environment Index derive from the data of the Seventh Population Census of China in Bengbu City and the point of interest (POI) data of various facilities were collected from the map website. The “Seventh Population Census of China” refers to the national census conducted by China in 2020, and POI refers to the point data in the electronic map on the Internet, which basically consists of four attributes, namely, name, address, coordinates, and category. The 500-meter distance represents the daily 10-min walking distance for residents, with a 500-meter buffer zone encompassing the road network and POI density ranges (19).

### 2.3 Research framework

**Figure 1:**
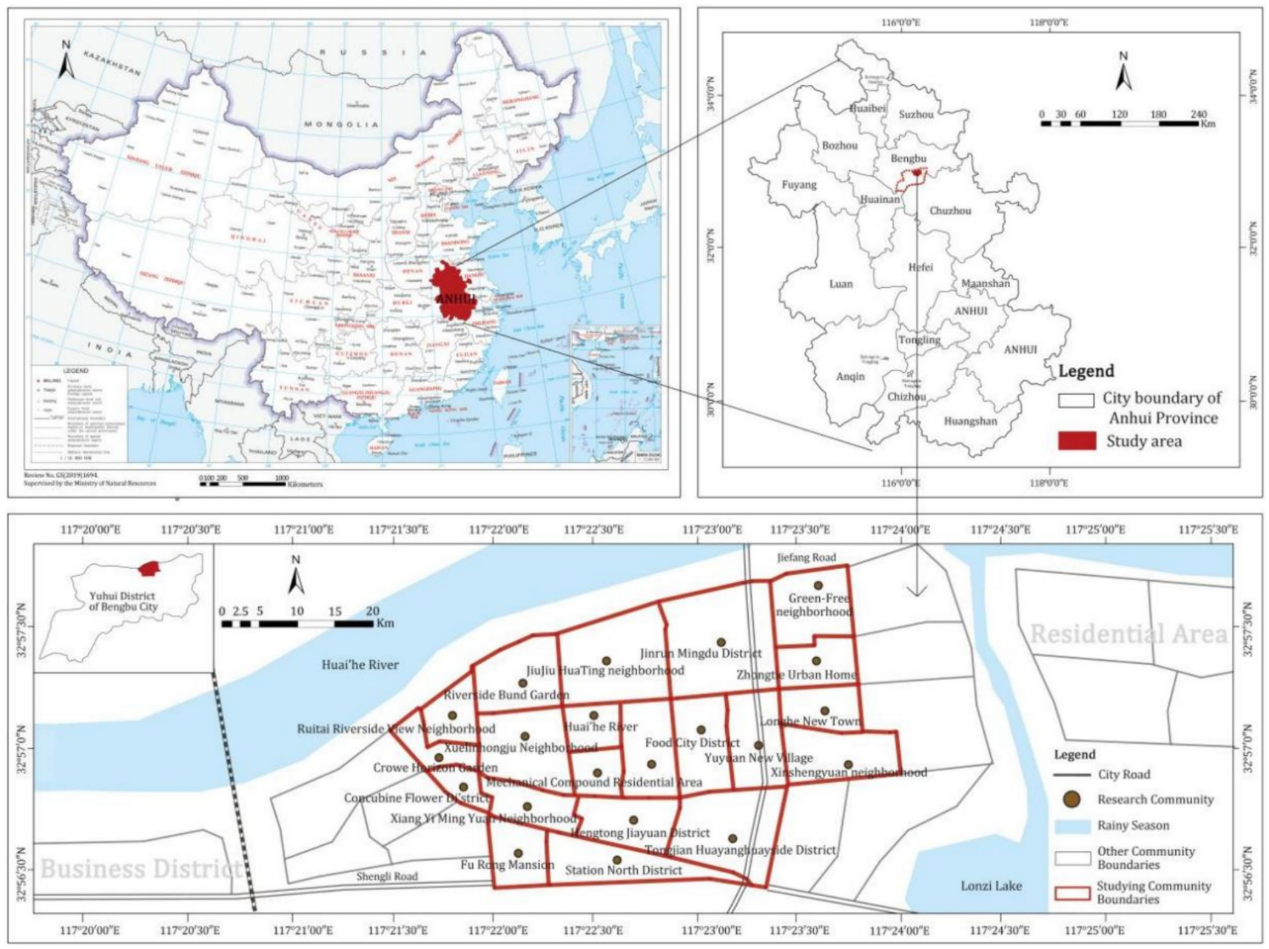
Distribution of the scope of investigation.

This article employs a statistical analysis of data pertaining to the relationship between the community’s built environment and hypertension among the residents. Furthermore, a multiple regression model was employed to ascertain the significance of the impact of the community’s built environment on hypertension and to investigate the manner in which the built environment influences hypertension levels through health behavior pathways. Finally, the improvement strategies are suggested. The theoretical analysis model is shown in Figure 1.

### 2.4 Data analysis

#### 2.4.1 Multiple regression model

**Figure 2:**
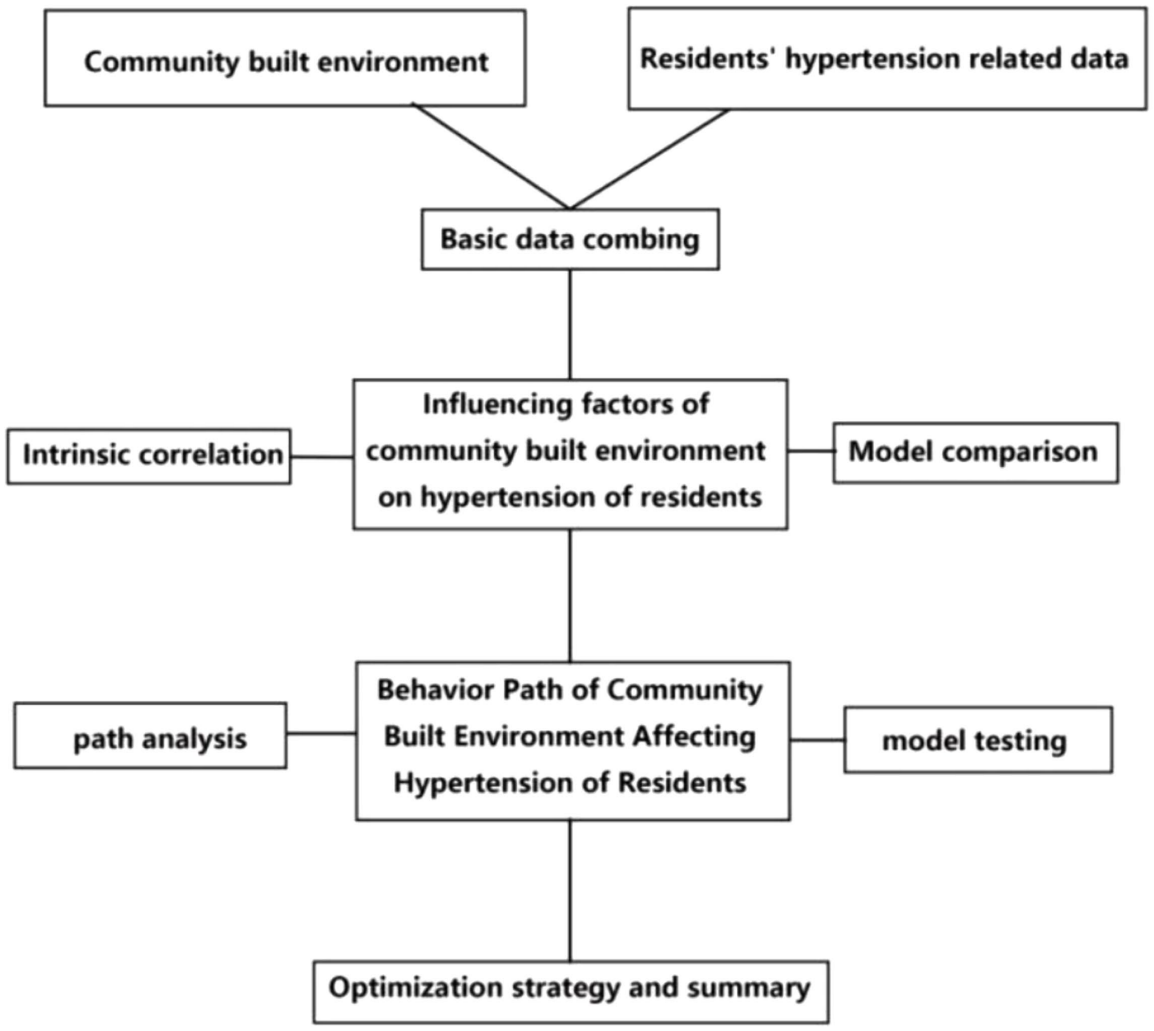
The influence of urban community’s built environment on residents’ hypertension and its path research framework.

In this article, the statistical control method (20) is selected for analysis. The hypertension level is usually influenced by many factors such as personal economic, social attributes, and built environment. Individual health status (including height, weight, and current year’s medical costs) and poor lifestyle habits (such as smoking and alcohol consumption) represent significant external factors influencing population health. It is therefore essential to incorporate these variables into the model and to evaluate the robustness of the regression results. The model is represented as follows:


(1) $$H_{\mathrm{it}}=\alpha +\beta_{\mathrm{BE}}BE_{\mathrm{it}}+\beta_{S}SES_{\mathrm{it}}+\beta_{S}HC_{\mathrm{it}}+\beta_{S}UNH_{\mathrm{it}}+\varepsilon$$

where H_it_ is the hypertension rank status of individual *i* living in community *t*. BE is the matrix of the community’s built environment variables of the study individual (the core variable of this study). SES is the matrix of socioeconomic attributes variables of the study individual (the control variable of this study). HC and UNH are the health status and bad habits, respectively, and α and ε are the constant and error terms of the regression model, respectively (Equation 1). In order to control for the effects of individual health status and adverse behavior, the model setting of previous studies (21) was employed. Subsequently, the hypothesis that the relationship between the built environment and the grade of hypertension remains statistically significant will be tested. This will enable us to ascertain whether the effects of hypertension are attributable to individual health status and poor behavior (22) (Figure 2).

#### 2.4.2 Mediation effect test

**Figure 3:**
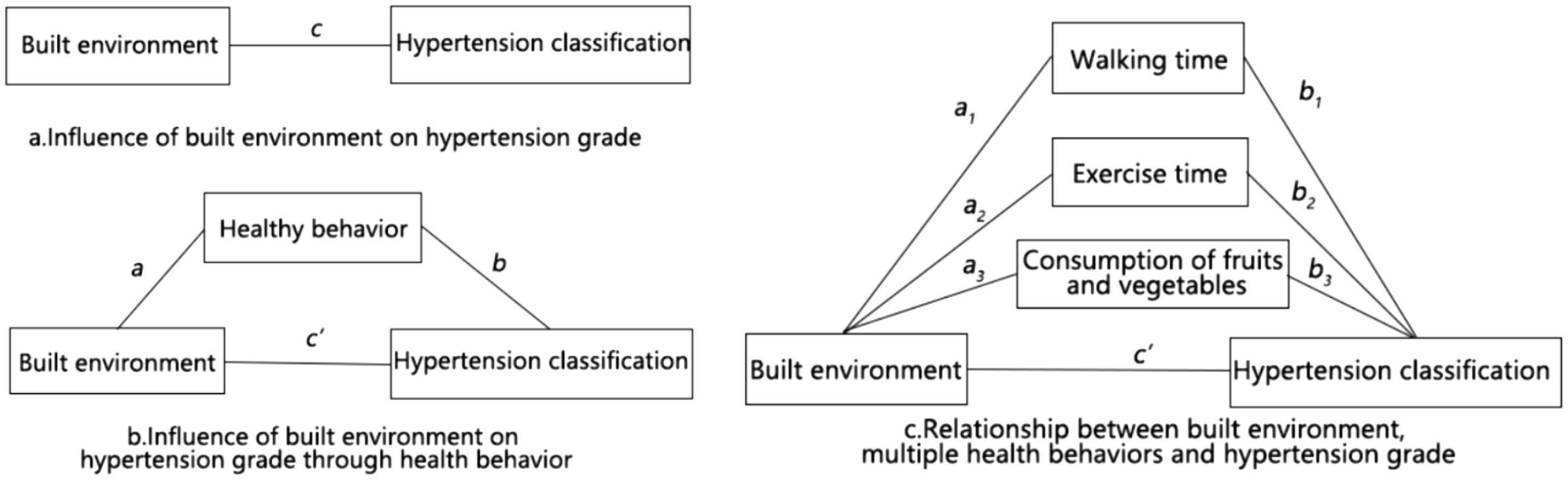
Relationship between the built environment, health behavior, and hypertension grade.

Figure 3 shows the relationship between the built environment, health behavior, and risk of hypertension. The total effect of the built environment on the risk of hypertension as calculated from the model *H_it_* is shown in Figures 3A,C. Given that the built environment exerts a partial influence on hypertension classification through the promotion of health-related behaviors, the total effect, designated as c, is classified into three distinct categories: a, b, and c’. In this context, a and b represent the effects of the total effect c through the health behavior pathway, while c’ denotes the effect of the other pathways. On the basis of Figure 3B, a structural equation model diagram of multiple mediators was formed (Figure 3C), and health behaviors were classified into three aspects: walking time, exercise time, and amount of fruits and vegetables consumed. The bias-corrected non-parametric percentile Bootstrap method was also used to test whether mediating effects a1b1, a2b2, and a3b3 were present in c.

#### 2.4.3 Handling of nested data structures

Model 1 incorporates a range of variables, including socioeconomic attributes and health status, which are measured on an individual scale. However, the community-built environment indicators are consistent for all the residents within the same community, and the respondents are not independent individuals. It was thus necessary to ascertain whether the community-built environment indicators exerted an influence on the regression model. Therefore, the null model of the multilevel model was constructed to test.


(2) $$H_{\mathrm{it}}=\alpha_{00}+\mu_{0t}+\varepsilon_{\mathrm{it}}$$

where α_00_ denotes the total mean of the hypertension classes in the sample, μ_0t_ denotes the random effect of the community, and ε is the error term. The intra-group correlation coefficient (ICC) was obtained by finding the variance on both sides of it, and if ICC < 0. 06, it indicates that the difference between groups is not significant (23) and can still be tested using model 1.

## 3 Results

### 3.1 Effect of urban built environment on hypertension of the population

As shown in Table 1, the overall regression of Model IV was statistically significant (*R*^2^ = 0. 118, *F* = 143. 99). In terms of model test indicators, the *R*^2^ value of Model III (0. 118) was higher than that of Models I (0. 077) and II (0. 108), and it can be assumed that the model with the inclusion of individual health status and bad habits was more effective (Table 1). Therefore, the subsequent study was conducted based on Model IV. Among the community’s built environment factors, population density (*β* = 0. 014), road network density (*β* = 0. 02), and supermarkets and convenience stores (*β* = 0. 033) positively predicted residents’ hypertension levels (*p* < 0. 05), indicating that some of the built environment factors positively affected residents’ hypertension levels, while park square (*β* = 0. 244*) and other built environment factors were less significantly associated with hypertension levels. Among the economic and social attributes of residents, age (*β* = 0. 013**) and marital status (*β* = 0. 117*) had a greater effect on hypertension levels, while gender (*β* = 0. 073) had a less effect on hypertension levels. In conclusion, regression analyses were employed to elucidate elements of the built environment, including neighborhood density, population density, and road density, as well as elements of personal attributes, such as residents’ gender and age. Furthermore, the analysis revealed that residents’ self-assessed health status and health behaviors exert a significant influence on hypertension levels. Among the personal health conditions, obesity (*β* = 0. 041**) and medical expenses (*β* = 0. 044**) had a greater impact on residents’ hypertension levels. Meanwhile, the effect size and statistical significance of each variable in the model remained unchanged. This indicates that the notable impact of the built environment on the hypertension grade of residents is more substantial when individual health status and unhealthy habits are not taken into account. The nestedness of the data was tested according to Equation 2, and ICC = 0. 034 (<0. 06), so the results of the regression model were still accurate in that the built environment in the city had little effect on individual independence.

**Table 1:** Influence of urban built environment on hypertension grade.

Variable	Define	Model 1	Model 2	Model 3	Model 4
Community built environment					
Population density	Population density in the community (unit: 10, 000 people /km^2^)	0.022 (0.015)	0.016 (0.015)	0.014 (0.015)	0.014 (0.009)
Road network density	Road network density (unit: km/km^2^)	0.031 (0.048)	0.024 (0.048)	0.021 (0.048)	0.02 (0.046)
Clinics and hospital	Density of clinics and hospitals (unit: one /km^2^)	−0.028* (0.013)	−0.03* (0.013)	−0.029* (0.013)	−0.028 (0.015)
Supermarket convenience store	Density of convenience stores in supermarkets (unit: one /km^2^)	0.028** (0.011)	0.033** (0.011)	0.033** (0.011)	0.033** (0.008)
Park square	Density of Park Square (unit: one /km^2^)	0.282** (0.083)	0.277** (0.083)	0.243** (0.083)	0.244* (0.086)
Economic and social attributes					
Gender	Men = 1; Women =0	0.075 (0.045)	0.078 (0.045)	0.074 (0.045)	0.073 (0.044)
Age	Age of respondents (Unit: Years)	0.014** (0.002)	0.012** (0.002)	0.013** (0.002)	0.013** (0.001)
Marital status	Married = 1; Other =0	0.153* (0.062)	0.126* (0.062)	0.117 (0.062)	0.117* (0.05)
Academic degree	Junior high school and above education = 1; Other =0	−0.142* (0.058)	−0.104 (0.058)	−0.113 (0.058)	−0.112 (0.066)
Monthly income^1^	Respondents’ personal monthly salary income^1^	0.043* (0.021)	0.039 (0.021)	0.018 (0.022)	0.018 (0.02)
Individual health status					
Fat	Respondents’ body mass index (unit: kg/m^2^)		0. 041** (0.006)	0. 041** (0.006)	0. 041** (0.009)
Medical expenses^2^	Respondents’ current medical expenditure^2^		0. 04** (0.015)	0. 044** (0.015)	0. 044** (0.014)
Bad habit					
Smoke	Respondents smoking = 1; Other =0			0.144* (0.056)	0.144 (0.077)
Drink	Respondents drinking = 1; Other =0			0. 109* (0.053)	0. 109 (0.066)
Constant term		0.021 (0.414)	−0.894* (0.43)	−0.966* (0.43)	−0. 973* (0.455)
*R* ^2^		0.077	0.108	0.118	0.118
*F* value		*F* (11, 1837) = 13.929 *p* = 0.000	*F* (13, 1770) = 16. 561 *p* = 0.000	*F* (15, 1751) = 15.489 *p* = 0.000	*F* (15, 20) = 143.99 *p* = 0.000

^1^This variable is an ordered categorical variable, below 1,000 RMB = 1, 1,000–2000 RMB = 2, 2000–3,000 RMB = 3, 3,000–4,000 RMB = 4, and above 4,000 RMB = 5. ^2^This variable is an ordered categorical variable, below 600 RMB = 1, 600–1,000 RMB = 2, 1,000–1,500 RMB = 3, 1,500–2000 RMB = 4, above 2000 RMB = 5; **p < 0. 05, *p < 0. 1.

Consequently, among the variables pertaining to the built environment, which have been constructed by the community, a negative association was observed between the density of clinics and hypertension class. Conversely, a positive association was identified between the densities of supermarket convenience store and park square, and hypertension class. Among the socioeconomic attribute variables, age was found to be negatively associated with hypertension class. Among the personal health status variables, there was a positive correlation between obesity and hypertension, and a negative correlation between healthcare costs and hypertension. Among the detrimental lifestyle habits, both smoking and alcohol consumption were found to be associated with an increased prevalence of hypertension.

### 3.2 Constructed pathways of environmental influences on the hypertension grade of the population

**Figure 4:**
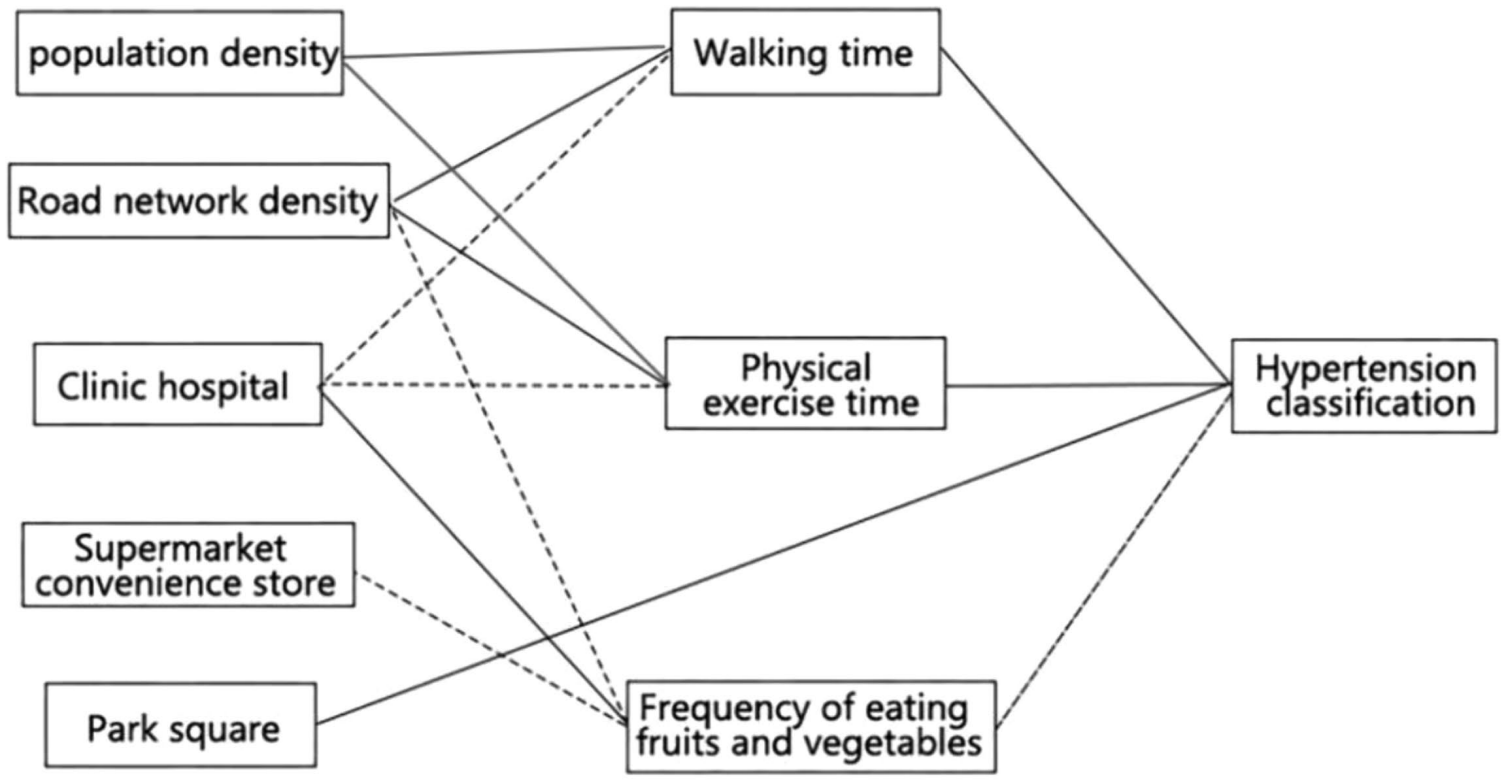
Influence of built environment on hypertension and its route summary. The solid line indicates that the sign of the coefficient is positive, and the dotted line indicates that the sign of the coefficient is negative.

The investigation revealed that diverse built environments exerted an impact on hypertension grades through three health behavior pathways, namely, walking time, physical activity time, and frequency of eating fruits and vegetables. In view of the influence of additional control variables on the mediation model, the control variables were removed, and the impact of the independent variable “built environment” on hypertension through the health behaviors was considered in isolation (Figure 4). In particular, walking time was found to be positively associated with population density and road network density, and negatively associated with clinic-hospital density. The time spent engaging in physical activity was found to be positively correlated with road network density and negatively correlated with clinic-hospital density. The frequency of fruit and vegetable consumption was found to be positively correlated with the density of medical facilities.

After analyzing the above model using SPSS, version 29.0 and Mplus 8.2, the study found that (1) the built environment affects the hypertension class by influencing the walking time of the residents. Table 2 shows that higher road network density and lower clinic-hospital density increase the risk of hypertension among the residents by increasing the residents’ walking time. Higher road network density may increase the risk of hypertension by having residents disturbed by street noise, resulting in stressful and depressing emotions. A lower density of clinics and hospitals may increase the time for residents to go to hospitals for regular medical checkups, thus reducing the likelihood that residents take the initiative to have medical checkups to reduce the chance of contracting the disease. (2) The built environment affects hypertension class by influencing the amount of time residents spend in physical activity. Table 3 demonstrates that higher road network density (c’ = 0. 054) and lower clinic-hospital density (*c*’ = −0. 034*) increase the risk of hypertension among residents by increasing the time they spent on physical activity. Higher road network density may increase residents’ activity time and activity intensity, increasing the cardiovascular burden and making residents’ blood pressure rise significantly in a short period of time. Lower clinic-hospital density may lead to the untimely popularization of antihypertensive knowledge, which does not play a good role in guiding residents to live a healthy life and thus increases the potential risk of hypertension. (3) The built environment affects hypertension levels by influencing the frequency of consumption of fruits and vegetables. Table 4 indicates that lower road network density (*c*’ = 0. 05), lower supermarket convenience store density (*c*’ = 0. 034**), and higher clinic-hospital density (*c*’ = −0. 032*) reduce the risk of hypertension among residents by increasing the frequency of the consumption of fruits and vegetables by the residents. Lower road network density reduces residents’ exposure to harmful gasses such as car exhaust. A lower density of supermarkets and convenience stores reduces the potential risk of hypertension by decreasing calorie intake and increasing fruit and vegetable intake. A higher density of clinics and hospitals helps residents to receive more timely treatment to control the incidence of hypertension before its onset.

**Table 2:** Influence of built environment on hypertension grade through walking time.

Item	c	a	b	a*b	a*b	*c*’
	Total effect			Mediating effect value	(95% BootCI)	Direct effect
Population density	0.031*	−0.047	0.002**	0	−0.004 ~ 0.003	0.031*
Road network density	0.064	8.695**	0.002**	0.013	0.004 ~ 0.028	0.05
Clinic hospital	−0.037**	−1.943**	0.002**	−0.003	−0.023 ~ −0.003	−0.034*
Supermarket convenience store	0.037**	0.045	0.002**	0	−0.005 ~ 0.005	0.037**
Park square	0.340**	3.255	0.002**	0.005	−0.001 ~ 0. 005	0.335**

*p < 0. 05, **p < 0. 01. Bold indicates that the mediation effect is significant.

**Table 3:** Influence of built environment on hypertension level through physical exercise time.

Item	c	a	b	a*b	a*b	*c*’
	Total effect			Mediating effect value	(95% BootCI)	Direct effect
Population density	0.031*	−0.112	0.001**	0	−0.004 ~ 0.003	0.031*
Road network density	0.064	8.587**	0.001**	0.01	0.002 ~ 0.023	0.054
Clinic hospital	−0.037**	−2.783**	0.001**	−0.003	−0.025 ~ −0.002	−0.034*
Supermarket convenience store	0.037**	0.861	0.001**	0.001	−0.001 ~ 0.010	0.036**
Park square	0.340**	7.346	0.001**	0.009	−0.000 ~ 0.007	0.332**

*p < 0. 05, **p < 0. 01. Bold indicates that the mediation effect is significant.

**Table 4:** Influence of the frequency of eating fruits and vegetables on hypertension level in built environment.

Item	c	a	b	a*b	a*b	*c*’
	Total effect			Mediating effect value	(95% BootCI)	Direct effect
Population density	0.021	−3.222	−0.001**	0.002	−0.002 ~ 0.010	0.019
Road network density	0.084	−62.770**	−0.001**	0.034	0.016 ~ 0.065	0.05
Clinic hospital	−0.043**	19.800**	−0.001**	−0.011	−0.070 ~ −0.018	−0.032*
Supermarket convenience store	0.039**	−7.975**	−0.001**	0.004	0.007 ~ 0.030	0.034**
Park square	0.359**	−12.355	−0.001**	0.007	−0.002 ~ 0. 007	0.352**

*p < 0. 05, **p < 0. 01. Bold indicates that the mediation effect is significant.

## 4 Discussion

Previous literature has explored the impact of the built environment on residents’ health from environmental perspectives such as compact use of urban space, functional mixing, and pedestrian friendliness. However, in the context of an aging population in old urban areas, there are fewer studies focusing on the impact of the built environment on the incidence of hypertension through the behavior of residents (24). Nowadays, in the face of population aging, it is of practical significance to study the impact of the built environment on hypertension, a common disease among the older adult (25).

In studying the effects of built environment on residents’ health, among the community’s built environment variables, clinic density was negatively associated with hypertension class. This is consistent with previous research findings (9), where the presence of more number of clinics provided residents with more opportunities for blood pressure measurement and health education, making it possible for residents to reduce the risk of hypertension in advance. The density of supermarkets and convenience stores was found to be positively associated with the grade of hypertension, which is also consistent with previous findings (10). In other words, the greater the density of supermarkets and convenience stores, the greater the likelihood that residents will consume foods high in oil and salt, and thus be more likely to develop hypertensive disorders. The positive correlation between park square density and hypertension classification differs from the findings of a regional study conducted in cities with a greater abundance of parks and greenery (11). In these cities, an increase in park square density may result in greater exposure to natural environments, which could contribute to a reduction in the incidence of hypertensive disorders. However, some scholars have also found that park squares may also have a risk of damaging health (26), and this study further validates their findings. Furthermore, the study area’s location in an older urban setting resulted in a relatively low number of parks per neighborhood (*M* = 0.239), accompanied by a high degree of clustering. This may result in a discrepancy between the findings of the study and the conclusion that an increased density of park squares is associated with an increased risk of hypertension (27, 28).

In addition, among the socioeconomic attribute variables, age was negatively associated with hypertension grade. This finding is consistent with previous studies (29), where the human cardiovascular system undergoes degenerative changes with age and increased blood pressure may lead to an increase in hypertension grade. Among the individual health status variables, obesity can lead to increased hypertension, while obese individuals have thicker subcutaneous fat and increased blood volume, leading to higher blood pressure (30). Higher medical expenses of residents will indirectly lead to the increase in hypertension risk, and higher medical expenses often mean that residents have low immunity or chronic diseases, which will lead to an increase of hypertension risk (31). Among the bad habit variables, both smoking and drinking increase the risk of hypertension, probably because harmful components in tobacco (32) and alcohol (33) cause damage to blood vessel walls, as well as other hazards, which increase the risk of hypertension. Therefore, in daily life, to strengthen health education for the residents, community workers should regularly publicize the dangers of hypertension and the knowledge on how to prevent it, such as reducing smoking and drinking (34, 35). At the same time, strengthening health education for teenagers in schools and developing good living habits from childhood will indirectly affect the future level of hypertension (36).

Higher road network density and lower clinic-hospital density can increase the risk of hypertension among residents by increasing the walking time. A higher density of road network increases the daily walking time of residents, which is in accordance with the previous research results (37). The dense road network shows that the streets are closely connected and plays an important role in facilitating residents’ walking habits (38). However, the study area is located in the old city, and longer outdoor walking time on both sides of the road will increase the possibility of being exposed to pollution (39, 40), which will increase the risk of hypertension (41). A reduction in the density of both clinics and hospitals has been found to result in an increase in the amount of time residents spend walking on a daily basis. This finding is consistent with the results of a previous study (42). In other words, a reduction in the number of healthcare service destinations results in a decrease in accessibility to treatment (43). This indicates that the daily journey to healthcare facilities becomes more onerous, thereby increasing the time residents spend walking per day.

Higher road network density and lower clinic hospital density increase the risk of hypertension for residents by increasing their physical exercise time. The increase in road network density increases the time of residents’ sports activities, which is consistent with the previous research results (44). Specifically, the increase of road network density improves the accessibility of sports venues, thus increasing the frequency of sports activities (45). A novel finding is that a reduction in clinic-hospital density in the study sample is associated with an increase in the frequency of physical activity among the residents. This may be attributed to the observation that the residents of communities with a reduced number of clinics and hospitals have diminished access to healthcare services (46), which may result in a greater propensity to engage in rigorous physical activity. In particular, residents with cardiovascular disease will engage in more unguided exercise. However, research shows that high-frequency exercise increases patients’ blood pressure and intensifies the risk of hypertension (47), especially when hypertensive patients exercise in a blind way (48).

Lower road network density and supermarket convenience store density but higher clinic hospital density would reduce the risk of hypertension among residents by increasing the frequency of eating fruits and vegetables. The frequency of eating fruits and vegetables had a significant negative association with hypertension grade under the influence of the built environment in older urban areas, which is consistent with previous studies (49). Vegetables and fruits are a rich source of essential nutrients, including dietary fiber and vitamins, which can play a role in the prevention of hypertension. Nevertheless, a reduction in the consumption of other food items with a high salt and fat content has been demonstrated to enhance immunity and safeguard the cardiovascular system, thereby reducing the likelihood of hypertension. A reduction in road network density has been observed to result in an increase in the frequency of fruit and vegetable consumption among the residents. This may be attributed to the observation that neighborhoods with lower densities of surrounding road networks typically exhibit superior environmental quality. A more favorable natural environment is conducive to the adoption of a healthier diet among the residents. Residents far from the city center will also grow some vegetables and fruits in their own yards, which will increase their consumption of fruits and vegetables and reduce their risk of chronic diseases (50). An increase in the density of clinics and hospitals is associated with a higher frequency of fruit and vegetable consumption among the residents. This may be attributed to the prevalence of health awareness slogans and health education talks in hospital clinics. This will facilitate an improvement in residents’ attitudes toward healthy eating, which will in turn result in an increase in the frequency of consumption of fruits and vegetables. An increase in the density of supermarket convenience stores decreases the frequency of eating fruits and vegetables among residents, which is in line with previous studies (14). Supermarket convenience stores will sell more high-calorie food, fast food, and other types of unhealthy food, which will make residents choose fewer fresh fruits and vegetables.

Therefore, the study area needs to optimize the corresponding community built environment and reduce the risk of hypertension by improving the density of road network, clinics, hospitals, supermarkets, convenience stores, and park squares. This article provides the following recommendations. (1) The road network needs to be improved. In the renewal of the old city, road network should be reorganized to reduce the relative walking time of residents on both sides of the road and increase other activities (51). (2) A high density of clinics and hospitals will reduce the risk of hypertension through residents’ behavior. It is desirable that land for clinics and hospitals be reserved at the beginning of planning, and it is advisable to gradually increase medical land and investment in urban renewal, and increase the current density through pilot planning of “smart health stations.” (3) In terms of supermarket convenience stores, more supermarket convenience stores will increase the risk of hypertension among residents’ who may consume less fruits and vegetables. Consequently, in future planning, some supermarket convenience stores should be transformed into vegetable and fruit markets and stores.

Since related research in the context of heavy aging in old urban areas is still in its infancy, and in view of the existing research on healthy cities, future empirical analyses on this issue can focus on the following aspects: (1) based on the residents’ traveling surveys, the spatial analysis scope that meets the actual activities of individuals can be delineated and the accuracy of the activity space and the amount of their activities can be improved and increased; (2) on the hypertension level of residents, the Internet open-source data and field survey data can be combined to test the similarities and differences between objective built environment indicators and subjective environmental perceptions; (3) the differences in the mechanism of action among different populations can be explored, with particular emphasis on the impact of the urban built environment on the health status of disadvantaged social classes.

## 5 Conclusion

Each of the built environment factors had a significant effect on the level of hypertension in the population, but were not interfered with by their individual health status or bad habits. A high density of supermarkets and convenience stores, parks and squares, and a low density of clinics and hospitals increase the risk of hypertension and are detrimental to residents’ health. The built environment will influence residents’ health-related behavior and affect their own hypertension levels. To be more specific, higher road network density and lower density of clinics and hospitals increase walking and physical exercise time and reduce the frequency of eating fruits and vegetables, thus increasing the risk of hypertension. Higher supermarket and convenience store densities increase the risk of hypertension by decreasing the frequency of fruit and vegetable consumption.

This article is based on cross-sectional survey data only, and future studies utilizing longitudinal survey data are needed to further explore the effects due to environmental changes, which will more accurately explain the relationship between the built environment and residents’ hypertension.

## Data Availability

The original contributions presented in the study are included in the article/supplementary material, further inquiries can be directed to the corresponding author.
